# Predicting the Impact of the 2011 Conflict in Libya on Population Mental Health: PTSD and Depression Prevalence and Mental Health Service Requirements

**DOI:** 10.1371/journal.pone.0040593

**Published:** 2012-07-13

**Authors:** Fiona J. Charlson, Zachary Steel, Louisa Degenhardt, Tien Chey, Derrick Silove, Claire Marnane, Harvey A. Whiteford

**Affiliations:** 1 School of Population Health, University of Queensland, Herston, Queensland, Australia; 2 School of Psychiatry, University of New South Wales, Prince of Wales Hospital, Randwick, New South Wales, Australia; 3 Centre for Health Policy, Programs and Economics, School of Population Health, The University of Melbourne, Carlton, Victoria, Australia; 4 National Drug and Alcohol Research Centre, University of New South Wales, Sydney, Randwick, New South Wales, Australia; Yale School of Public Health, United States of America

## Abstract

**Background:**

Mental disorders are likely to be elevated in the Libyan population during the post-conflict period. We estimated cases of severe PTSD and depression and related health service requirements using modelling from existing epidemiological data and current recommended mental health service targets in low and middle income countries (LMIC’s).

**Methods:**

Post-conflict prevalence estimates were derived from models based on a previously conducted systematic review and meta-regression analysis of mental health among populations living in conflict. Political terror ratings and intensity of exposure to traumatic events were used in predictive models. Prevalence of severe cases was applied to chosen populations along with uncertainty ranges. Six populations deemed to be affected by the conflict were chosen for modelling: Misrata (population of 444,812), Benghazi (pop. 674,094), Zintan (pop. 40,000), displaced people within Tripoli/Zlitan (pop. 49,000), displaced people within Misrata (pop. 25,000) and Ras Jdir camps (pop. 3,700). Proposed targets for service coverage, resource utilisation and full-time equivalent staffing for management of severe cases of major depression and post-traumatic stress disorder (PTSD) are based on a published model for LMIC’s.

**Findings:**

Severe PTSD prevalence in populations exposed to a high level of political terror and traumatic events was estimated at 12.4% (95%CI 8.5–16.7) and was 19.8% (95%CI 14.0–26.3) for severe depression. Across all six populations (total population 1,236,600), the conflict could be associated with 123,200 (71,600–182,400) cases of severe PTSD and 228,100 (134,000–344,200) cases of severe depression; 50% of PTSD cases were estimated to co-occur with severe depression. Based upon service coverage targets, approximately 154 full-time equivalent staff would be required to respond to these cases sufficiently which is substantially below the current level of resource estimates for these regions.

**Discussion:**

This is the first attempt to predict the mental health burden and consequent service response needs of such a conflict, and is crucially timed for Libya.

## Introduction

International attention has focused on the civil conflict in Libya following anti-government protests starting in February 2011 which led to the fall of the regime led by Muammar Gaddafi. The events in Libya were associated with widespread violence and NATO military intervention under United Nations Security Council resolution 1973 adopted on 17 March 2011. [1] The scale of physical casualties associated with the conflict have been recently documented, [2] with commentators identifying the need for humanitarian agencies to consider mental as well as physical health in responding to the aftermath of this complex emergency. [3] Estimates of trauma-related physical morbidity have been made, [4] but there is no quantitative assessment of the likely mental health and related psychosocial consequences of the conflict.

Evidence indicates that exposure to conflict-related potentially traumatic events (PTE) will lead to an elevation in the prevalence of mental disorders, including depression and post-traumatic stress disorder (PTSD), among exposed sections of the Libyan population. [5]–[8] The disability and burden associated with these mental disorders have been well-documented, [9] as has their socio-economic impact. [10] Steel and colleagues have published a recent systematic review and meta-regression of depression and PTSD prevalence in conflict affected populations [11]. This meta-regression enables the estimation of projected mental disorder prevalence for conflict-exposed populations stratified according to risk exposure variables. Key stratifying variables include: the level of “political terror” as defined by the political terror scale (PTS) a country level measure of political violence compiled from human rights reports; [12] a potentially traumatic event (PTE) adversity ratio derived from the level of exposure to PTE’s recorded within the samples included in the systematic review; time since conflict, with closer proximity to the traumatic events resulting in higher rates of mental disorder; and whether populations were displaced due to the conflict. [11].

The increased prevalence of mental disorders in combination with the limited health resources available in low- and middle-income countries (LMIC’s) highlights the need for health service planning to focus on the subgroup of severe cases that are most likely to benefit from access to treatment. [13] Mental health workforce requirements for LMIC’s have been modelled for eight priority mental, neurological and substance use disorders as part of the World Health Organisation (WHO) Mental Health Gap Action Programme (mhGAP).[13]–[15] When combined with an assessment of existing services and resources in a country, it is possible to identify potential service gaps, information that then informs funding decisions and program development. Country level information is collected annually by the WHO Department of Mental Health and Substance Abuse on mental health and neurology services, including information relating to the health and mental health workforce and infrastructure. [16], [17].

Studies that have been undertaken in post-conflict settings provide valuable datasets allowing us to estimate the prevalence of mental disorder in conflict zones. The present study represents the first attempt to draw on existing datasets to estimate the increase in numbers of people with mental disorders using a contemporary conflict exposed setting. We also apply a benchmark model of target setting [14] for mental health service requirements in LMIC’s_ENREF_14 and compare these requirements against those which already exist in Libya, as reported by WHO.

Our findings aim to be of assistance to conflict and post-conflict countries, including Libya, and the international health and humanitarian community in meeting the challenges of post-conflict reconstruction. [3] We also aim to address priorities for research identified within the Grand Challenges in Global Mental Health initiative [18]; specifically Goal A, identifying root causes, risk and protective factors; and one of the “top 25” challenges, of understanding the impact of poverty, violence, war, migration and disaster.

This paper aims to:

1. derive best-estimates for depression and PTSD prevalence in conflict-affected Libyan populations based on the factors known to influence the prevalence of these disorders;
2. estimate the number of severe cases and comorbidity between these disorders for given populations;
3. describe the mental health service requirements that would be needed in Libya to meet the needs of this population during the post-conflict period and compare this with the current service capacity in Libya.

## Methods

### Predicting Prevalence Estimates Following Conflict According to Key Conflict-related Variables

**Figure 1:**
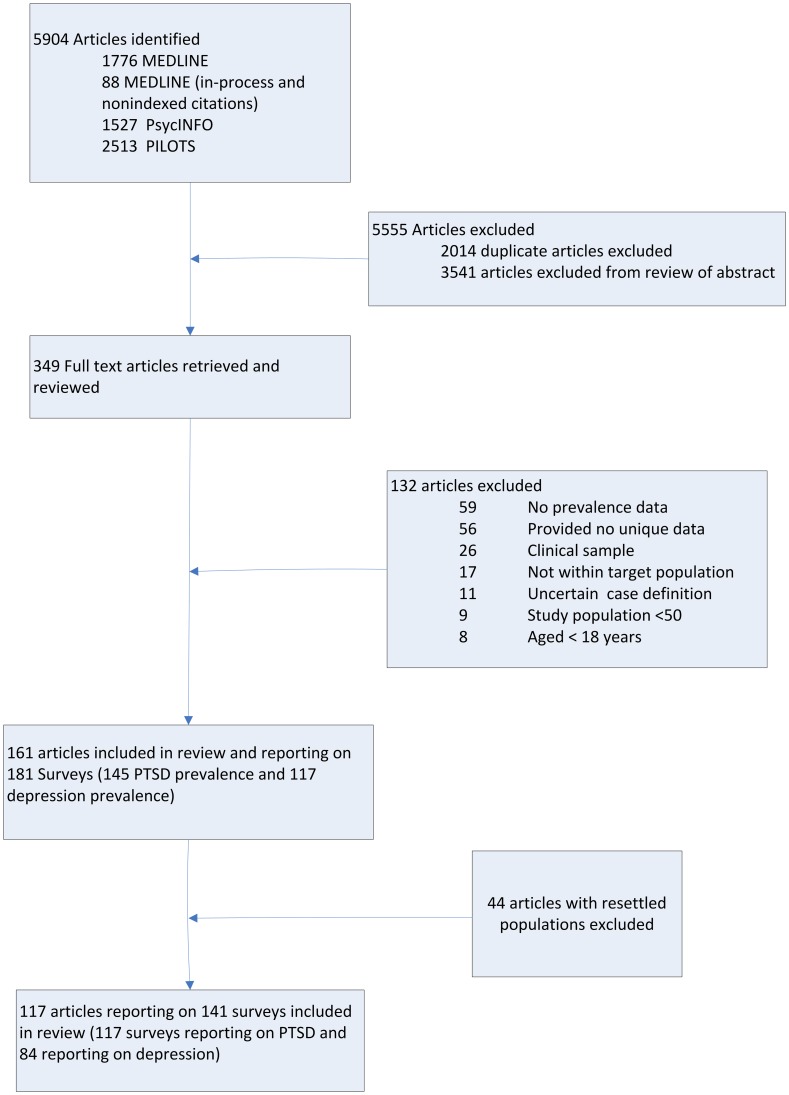
Systematic search flow diagram.

Post-conflict prevalence estimates have been derived from models based on a previously conducted systematic review and meta-regression. The methodology has been described in detail elsewhere. [11] The supporting PRISMA checklist is available as supporting information; see Checklist S1. In brief, a systematic review of English language articles reporting the prevalence of depression or PTSD among displaced and conflict-affected populations published between 1980 and May 2009 was undertaken. The search followed the Meta-analysis of Observational Studies in Epidemiology reporting guidelines. [19] MEDLINE and PsycINFO were utilised to identify published articles reporting the prevalence of depression or PTSD among refugee, conflict-affected populations, or both. Mental health content was identified using terms *mental health* and *mental disorders* [*20*]; articles with refugees were identified using key words *refugees*, *war*, *genocide*, *holocaust*, *terrorism*, or *torture*. PILOTS bibliographic index was also searched using key words: *refugees, asylum seekers, displaced persons, internally displaced person, genocide, holocaust, persecution, torture*. The review identified 161 articles reporting data for 181 surveys, including 145 detailing PTSD prevalence and 117 depression prevalence. For the current analysis the prevalence estimates from surveys of refugee populations resettled to high income country settings included in the original meta-regression were omitted so as to more accurately reflect conflict exposed populations still resident in the source country or internally displaced that are more comparable to the situation in Libya. This reduced the number of articles to 117, consisting of 117 surveys reporting on PTSD and 84 surveys reporting on depression (Figure 1) (see Table S1 for a summary of included studies).

Data extraction included methodological characteristics that were likely to affect prevalence rates, including study sample size and sampling method, and substantive study population characteristics, such as sociodemographic characteristics, place of survey, exposure to torture and other PTE’s, PTS score, residency status, and time since the cessation of major hostilities.

The PTS provides two separate measures of the political terror experienced in a country during a given year based on independent qualitative narrative reports produced by the United States Department of State and Amnesty International. It is considered the most comprehensive and most accurate index of its type and hence was chosen in favour of other potential measures of political terror within a population. [21] Libya received a rating of 3 for the majority of the past two decades, being described as a country in which “there is extensive political imprisonment…. execution or other political murders and brutality may be common. Unlimited detention, with or without a trial, for political views is accepted”. Since the rating for 2010 was not available at the time of writing, we estimated it to be greater than or equal to 4 based on the criterion - “Civil and political rights violations have expanded to large numbers of the population. Murders, disappearances, and torture are a common part of life. In spite of its generality, on this level terror affects those who interest themselves in politics or ideas”. [12], [22].

A strong dose-response association between exposure to PTE and both PTSD and depression has been reported across a wide number of individual surveys undertaken in the post-conflict literature [23]–[25]. We developed the PTE adversity ratio defined as the average number of PTE’s endorsed by a study sample divided by the total number of PTE’s assessed within a study as a measure of relative exposure to PTE. The PTE adversity ratio was developed in order to standardise the variation in the number of traumatic events assessed across surveys. Time since conflict, or end of major hostilities, is guided by information provided by the study authors or by reference to the PTS.

Meta-regression models were calculated in SAS version 9.1.3 [26] to examine sequentially the association of methodological and substantive factors with reported rates of PTSD and depression. In all models prevalence rates were transformed into logits. The Delta method was applied to compute within-study variance, namely, var(logit) = 1/case1+1/non_case. The models applied a mixed model with fixed and random- effects components. Regression logits were back transformed and expressed as absolute prevalence estimates. Methodological variables which demonstrated statistical significance in univariate analysis were included in the baseline methodological model. Associations of substantive factors were then assessed whilst adjusting for the baseline model and a series of regression models provided predicted prevalence estimates related to stratifications of the substantive factors. All tables present the additive variance for each substantive model above the variance accounted for by the baseline methodological model.

When choosing the most appropriate model for estimating prevalence in a given population, the rating on the political terror scale; intensity of exposure to traumatic events (PTE adversity ratio) and time in years since the cessation of major hostilities were selected as stratifying variables as they are standardised measures with high utility that had been identified as the most significant predictors of global prevalence [11], and have direct relevance to the conflict in Libya. While the population prevalence of torture has also been identified as a major global determinant of post-conflict mental disorder, there was insufficient information available at the time of writing to estimate the role of this form of abuse in relation to the exposed populations in Libya.

Model stratification was conducted according to ‘moderate’ (PTS <4) and ‘high’ (PTS ≥4) political terror, and ‘moderate’ (PTE adversity ratio <0.3) and ‘high’ (PTE adversity ratio ≥0.3) trauma. PTS and PTE adversity ratio cut-off points were derived from the thresholds associated with elevated risk for mental disorder identified in the full sample meta-regression analysis. The reference group to which all other models were compared was the moderate trauma and moderate political terror scenario. In addition, we have modelled the time effect of 3 years since the end of the conflict representing the projected notional end of the acute post-conflict phase.

### Selecting Key Libyan Populations

Populations in areas documented as high conflict or who have fled such areas were deemed the most likely to experience higher levels of exposure to PTE’s and ongoing risk of conflict related mental disorder. Six populations were identified as being profoundly affected by the conflict, based on assessments of documented reports from multiple humanitarian actor websites and other sources deemed as credible[2], [27]–[31] and form the basis of the modelling reported. Using this information, we further classified the regions according to the intensity of conflict. Displaced populations and those where the majority of the population are likely to have been exposed to high levels of trauma were categorised as ‘high intensity conflict’ areas. The cities of Misrata and Benghazi were epicentres of the conflict, but as large metropolitan areas, wide-reaching exposure to full intensity combat was limited. The majority of the population in these cities were, however, exposed to prolonged shelling and gunfire and hence they were classified as ‘medium intensity conflict’ areas.

1. Misrata (medium intensity conflict): the former opposition-controlled city of Misrata has been highlighted as a conflict zone [30]
2. Benghazi (medium intensity conflict): Benghazi was the main focus of the demonstrations against Colonel Gaddafi's rule with numerous reports of government troops opening fire on civilians resulting in substantial deaths [27]
3. Tripoli/Zlitan (high intensity conflict): Tripoli and displacement sites in former government controlled areas near Misrata (Zlitan and Al Khums) and the Nafusa Mountain area of Gharian have been highlighted by the United Nations High Commissioner for Refugees (UNHCR) as populations affected by widespread conflict [30]
4. Misrata displaced (high intensity conflict): within Misrata city itself there is a population displaced from their homes as a direct result of the state of terror [30]
5. Zintan (high intensity conflict): is the largest town in the Nafusa Mountains of western Libya which has suffered frequent rocket attacks affecting civilian homes and public areas. Most of its population fled and many live as refugees in Tunisia [28]
6. Ras Jdir (high intensity conflict): refugee camps on the border of Libya and Tunisia which largely hold migrant workers waiting for repatriation have been overloaded with those fleeing the violence in Libya. Camps are severely over capacity and conditions are very poor. [31]

Population size estimates were obtained preferentially from government and other official websites, such as those provided by the United Nations (UN); however unofficial websites were accepted where other sources were lacking. In the absence of a publicly available official population estimate but where we were able to identify multiple unofficial sources we took the average. The total population of Libya is 6,730,000. [32] The six areas modelled have a combined population of 1,236,600, approximately 20% of the national population.

### Estimating “Severe” Cases of PTSD and Depression

Numbers needing treatment and mental health service requirements in this paper are based on the estimated number of severe cases as these individuals are most likely to experience the highest levels of ongoing psychiatric disability. Severity proportions were based on data for affective and anxiety disorders recorded by the World Mental Health Survey (WMHS) Collaboration with anxiety disorder accepted as a proxy for PTSD. Severity ratings within the WMHS collaboration were derived from the Sheehan Disability Scale (SDS) [33] which measures impairment specific to a focal disorder. The score from the highest domain of impairment was selected to represent the category of severity for the prevalent disorder across WMHS studies. Numbers of 30-day cases which fell within an SDS score of 0–3 were categorized as mild, those 4–6 categorised as moderate and 7–10 as severe. Score cut-offs were based on recommendations by the WMHS Collaboration. [33], [34] Proportions of mild, moderate and severe cases, summing to a total 100% of cases were provided by age range and sex for each world region. MetaXL software version 0.1 [35] was used to calculate the pooled proportion of cases by severity category using a random effects model. Estimates of prevalence, 95% confidence intervals and severe case proportions were applied to the chosen populations to calculate the estimated number of severely impaired cases along with uncertainty ranges.

### Comorbidity

The proportion of PTSD cases comorbid with depression derived from general population and post conflict settings was estimated at 50%. [36], [37] Proportions of PTSD alone, depression alone and comorbid cases were extrapolated for disorder specific modeling.

### Predicting Mental Health Service Requirements for PTSD and Depression

Models of mental health service requirements for depression are taken from mental health cost-effectiveness studies for LMIC’s._ENREF_14 Chisholm and colleagues have estimated resources required to scale-up delivery of an essential mental healthcare package for treatment of four mental disorders (schizophrenia, bipolar disorder, depression and hazardous alcohol use) over a 10-year period. [13] This analysis has since been further developed, now highlighting health workforce gaps for eight mental and substance abuse disorders in LMIC’s. [14] The authors propose targets for service coverage, resource utilisation and healthcare staffing for treating major depressive disorder which were derived from published need assessments and economic evaluations and finalised in consultation with the Lancet Global Mental Health Group. We use these targets to estimate numbers of patients accessing and full-time equivalent staff requirements for a mental healthcare system (inpatient, outpatient and primary healthcare, and day care service) based on a suggested overall service coverage target of 33% of severe depression cases.

As there is currently no published mental health service requirement model for PTSD or comorbid PTSD/depression, the model was adapted to cover all cases we have estimated in our paper. Service types for PTSD were limited to treatment within the primary healthcare and psychosocial therapy setting in accordance with guidelines for the management of PTSD. [38] Service coverage targets were adjusted to 40% for comorbid cases of severe PTSD and severe depression, and 20% for severe PTSD-only cases as determined by expert consultation.

Clinician resources for day care services, outpatient/primary care visits, long-stay inpatients and acute inpatients were computed separately for each of the six populations. Full-time equivalent (FTE) is a unit to measure employed persons or students in a way that makes them comparable although they may work or study a different number of hours per week. FTE for primary healthcare and outpatient services were estimated based on the following algorithm; FTE = (Population * % prevalence * % severity * % coverage * Number of visits per case)/(11 consultations per day * 225 working days) [14], [39]. Where *% severity* refers to the percentage of all cases in the population that are severe (53% for depression as per above), and *% coverage* is a percent of those severe cases (33% for depression as modelled by Chisholm et al [13]). Number of visits per case were estimated by multiplying the % using a service by the average use of a service user, e.g. for depression day care this is 1% * 50 = 0.5 day care visits per average case. To estimate the full-time-equivalent staff required to meet inpatient service targets, Chisholm and colleagues used estimated bed–days as the starting input. To calculate the number of full-time-equivalent inpatient staff needed, staff:bed ratios for LMIC’s extracted from the literature were multiplied by the targeted number of inpatient beds. [14].

The total numbers of FTE staff for each level of service in each population were apportioned across health worker types. [13] FTE staff were categorised as medical (psychiatrists and medical officers), nurses (psychiatric nurses and general nurses) and psychosocial care providers (psychologists, social workers, occupational therapists, counsellors and other allied health workers).

To assess the gap in mental health service requirements based on estimates derived from the meta-regression models reported herein and the resource capacity in Libya we draw on data recording mental health resources in Libya compiled by WHO during 2005. [16].

## Results

The results of the meta-regression models for PTS, PTE adversity ratio and time since conflict are presented in Table S2. All models were statistically significant above the base methodological model (which adjusted for study sampling characteristics and type of diagnostic assessment (self report measure/structured diagnostic interview)). The exclusion of resettled refugee populations from the analysis led to an increase in the level of association with the PTS scale in relation to previously reported results for PTSD and depression. [11] Across all models, increased risk exposure as reflected in higher PTE scores, higher levels of political terror, or closer proximity in time to the conflict, was associated with increased prevalence of PTSD and depression.

### PTSD

Overall, the model in which included variables PTS and PTE adversity ratio accounted for the largest proportion of inter-study variability in PTSD prevalence rates (35.3%); 17.0% variance was explained by the methodological variables and a further 18.3% from the substantive variables (PTS and PTE adversity ratio). According to this model, PTSD prevalence amongst populations with a high level of political terror and a high level of PTE exposure was estimated to be 41.3% (95%CI 28.3–55.6). Lower levels of exposure to trauma (PTE adversity ratio <0.3) would equate to a prevalence of 32.3% (95%CI 18.2–50.6) (Table 1).

**Table 1:** Models of prevalence estimates for PTSD and depression during high political terror and stratified by exposure to potentially traumatic events.

	Odds ratio (95%CI)†	Total case prevalenceestimate (%) (95%CI)	Severely impaired case prevalence estimate (%) (95%CI)
**PTSD** ‡			
Moderate trauma	2.16 (1.01–4.64)*	32.3 (18.2–50.6)	9.7 (5.5–15.2)
High trauma	3.18 (1.79–5.68)*	41.3 (28.3–55.6)	12.4 (8.5–16.7)
**Depression** ‡			
Moderate trauma	2.61 (1.22–5.56)*	34.5 (19.8–52.9)	18.3 (10.5–28.0)
High trauma	2.95 (1.79–4.87)*	37.3 (26.5–49.6)	19.8 (14.0–26.3)

Abbreviations: PTSD = Post-traumatic stress disorder; Low political terror = PTS <4; High political terror = PTS ≥4; Moderate trauma = PTE adversity ratio <0.3; High trauma = PTE adversity ratio ≥0.3;

† reference category = moderate trauma*low political terror;

‡ All substantive predictor models are adjusted for significant methodological predictors (sample size and type of measure);

* Statistically significant OR compared to reference group.

Based on the pooled PTSD disability ratings from the World Mental Health Survey studies, 30.0% (95%CI 27.0–33.0) of PTSD cases fell into the severe disability range. [40] Hence, population projections for the total number of severe cases of PTSD in the model suggest that PTSD prevalence could range from 500 in Ras Jdir refugee camps housing 3,700 displaced Libyans at the time of writing, to approximately 65,000 in Benghazi in a population of 674,000 persons. Zintan, a relatively small mountain town that has been highly affected by conflict and which has the ability to provide only basic healthcare, [28] would have an estimated 5,000 cases of PTSD within the severely disabled range amongst a population of 40,000 (Table 2).

**Table 2:** Projections of severely impaired cases for various Libyan populations during a high state of political terror (rounded to nearest 100).

Population	Severely impaired caseprevalence estimate (%) (95%CI)	Total population	Estimated number of severely impaired cases (95%CI)
**PTSD**			
Moderate trauma settings			
Misrata [42], [43], [44], [45]	9.7 (5.5–15.2)	444,812	43,100 (24,500–67,600)
Benghazi [44], [45], [46], [47]	9.7 (5.5–15.2)	674,094	65,400 (37,100–102,500)
High trauma settings			
Tripoli/Zlitan [30]	12.4 (8.5–16.7)	49,000	6,100 (4,200–8,200)
Misrata displaced [30]	12.4 (8.5–16.7)	25,000	3,100 (2,100–4,200)
Zintan [28]	12.4 (8.5–16.7)	40,000	5,000 (3,400–6,700)
Ras Jdir camps [30]	12.4 (8.5–16.7)	3,700	500 (300–600)
Total		1,236,606	123,200 (71,600–182,400)
**Depression**			
Moderate trauma settings			
Misrata [42], [43], [44], [45]	18.3 (10.5–28.0)	444,812	81,400 (46,700–124,500)
Benghazi [44], [45], [46], [47]	18.3 (10.5–28.0)	674,094	123,400 (70,800–188,700)
High trauma settings			
Tripoli/Zlitan [30]	19.8 (14.0–26.3)	49,000	9,700 (6,900–12,900)
Misrata displaced [30]	19.8 (14.0–26.3)	25,000	5,000 (3,500–6,600)
Zintan [28]	19.8 (14.0–26.3)	40,000	7,900 (5,600–10,500)
Ras Jdir camps [30]	19.8 (14.0–26.3)	3,700	700 (500–1,000)
Total		1,236,606	228,100 (134,000–344,200)

PTSD = Post-traumatic stress disorder; Moderate trauma = PTE adversity ratio <0.3; High trauma = PTE adversity ratio ≥0.3.

### Depression

The meta-regression model that included PTS and the PTE adversity ratio accounted for 62.0% of overall inter-study variance. 46.5% was accounted for by the methodological model with 15.5% accounted for by PTS and PTE adversity. Depression estimates were high for all political terror categories, 34.5% (95%CI 19.8–52.9) and 37.3% (95%CI 26.5–49.6) for moderate and high trauma respectively (Table 1).

Findings from WMHS studies used as the basis for the current analysis indicate that 53.0% (95%CI 46.0–60.0) of depression cases had disability scores within the severe category. [41] Population projections of the number of total severe cases of depression based on these estimates range from 700 in Ras Jdir refugee camps to 123,400 in Benghazi (Table 2).

Taken across the six regions, the predictive models estimate a post-conflict total of 123,200 (71,600–182,400) cases of severe PTSD and 228,100 (134,000–344,200) cases of severe depression in an exposed population of approximately 1.24 million persons (Table 2).

### Comorbidity

Applying a comorbidity estimate of 50% to the current projections would identify 61,600 of the PTSD cases as being comorbid with major depression leading to an estimate of non-comorbid depression and PTSD cases of 43,300 and 61,600, respectively.

### Medium Term Projections

We also used the meta-analytic models to estimate prevalence within post-conflict settings of 3 or more years since the cessation of major hostilities, in which a more stable, low trauma environment may have had an opportunity to develop. The model yields an estimate of PTSD prevalence of 5.0% (95%CI 3.2–7.7) when considering only the more severely impaired and 9.0% (95%CI 5.9–13.0) for severe depression (Table 3) (see Table 4 for case projections). If a more complex environment emerges in which sections of the population remain at risk of high exposure to traumatic events, PTSD prevalence reduces; however, depression prevalence does not change from the current projections for the acute phase during high political terror (Table 3).

**Table 3:** Models of prevalence estimates for PTSD and depression after 3 years since the cessation of major conflict and stratified by level of exposure to potentially traumatic events.

	Odds ratio (95%CI)	Total case prevalenceestimate (%) (95%CI)	Severely impaired case prevalence estimate (%) (95%CI)
**PTSD** ‡			
Moderate trauma†	1.00	16.8 (10.7–25.6)	5.0 (3.2–7.7)
High trauma	1.55 (0.77–3.13)	23.9 (13.5–38.8)	7.2 (4.1–11.6)
**Depression** ‡			
Moderate trauma†	1.00	16.9 (11.2–24.6)	9.0 (5.9–13.0)
High trauma	2.93 (1.54–5.60)*	37.3 (23.8–53.2)	19.8 (12.6–28.2)

Abbreviations: PTSD = Post-traumatic stress disorder;

† reference category;

‡ All substantive predictor models are adjusted for significant methodological predictors (sample size and type of measure); Moderate trauma = PTE adversity ratio <0.3; High trauma = PTE adversity ratio ≥0.3;

* Statistically significant OR compared to reference group.

**Table 4:** Projections of severely impaired cases for various Libyan populations after 3 years since cessation of conflict (rounded to nearest 100).

Population	Severely impaired caseprevalence estimate (%) (95%CI)	Total population	Estimated number of severelyimpaired cases (95%CI)
**PTSD**			
Moderate trauma settings			
Misrata [42], [43], [44], [45]	5.0 (3.2–7.7)	444,812	22,400 (14,300–34,200)
Benghazi [44], [45], [46], [47]	5.0 (3.2–7.7)	674,094	34,000 (21,700–51,800)
High trauma settings			
Tripoli/Zlitan [30]	7.2 (4.1–11.6)	49,000	3,500 (2,000–5,700)
Misrata displaced [30]	7.2 (4.1–11.6)	25,000	1,800 (1,000–2,900)
Zintan [28]	7.2 (4.1–11.6)	40,000	2,900 (1,600–4,700)
Ras Jdir camps [30]	7.2 (4.1–11.6)	3,700	300 (50–400)
Total		1,236,606	64,800 (40,600–99,600)
**Depression**			
Moderate trauma settings			
Misrata [42], [43], [44], [45]	9.0 (5.9–13.0)	444,812	39,800 (26,400–58,000)
Benghazi [44], [45], [46], [47]	9.0 (5.9–13.0)	674,094	60,000 (40,000–87,900)
High trauma settings			
Tripoli/Zlitan [30]	19.8 (12.6–28.2)	49,000	9,700 (6,200–13,800)
Misrata displaced [30]	19.8 (12.6–28.2)	25,000	4,900 (3,200–7,000)
Zintan [28]	19.8 (12.6–28.2)	40,000	7,900 (5,000–11,300)
Ras Jdir camps [30]	19.8 (12.6–28.2)	3,700	700 (500–1,000)
Total		1,236,606	123,500 (81,300–179,100)

PTSD = Post-traumatic stress disorder; Moderate trauma = PTE adversity ratio <0.3; High trauma = PTE adversity ratio ≥0.3.

### Mental Health Service Coverage Targets

Based on the coverage target of 33% for major depression identified by Chisholm *et al*, [13] in LMIC settings, the numbers of severely depressed, non-comorbid patients requiring treatment would be approximately 14,000 across all six populations. Applying target coverage of 40% for comorbid cases of depression and PTSD and 20% for severe PTSD-only cases would result in an additional treatment requirement of approximately 24,000 and 12,000, respectively, yielding a total estimate of 50,000 persons affected by highly disabling manifestations of depression and PTSD who require access to mental health care.

The service coverage model proposed by Chisholm *et al* [13] for depressive disorders proposes that a high proportion of these patients could be effectively managed at the primary healthcare level using a range of evidence based treatments and that 20% of all cases could ideally require outpatient psychosocial therapies. It is proposed that all noncomorbid, PTSD patients can be managed within primary care and psychosocial services; however, a significant number of severely depressed and comorbid PTSD cases will require hospital outpatient services and a minority may require inpatient and daycare services (Table 5).

**Table 5:** Mental health service coverage targets and estimated number of severely impaired patients using various services for all six populations (total population  = 1,236,600).

	Depression alone(n = 43,300)	PTSD alone (n = 61,600)	Comorbid (n = 61,600)
**Service coverage targets (%)** 1			
Community residential (long-stay)^3^	0.5	-	1
Community psychiatric (acute care)^3^	2	-	4
Day care services^4^	1	-	1
Hospital out-patient service^4^	20	-	20
Primary healthcare – treatment^4^	30	10	40
Psychosocial treatment^4^	20	20	20
**Number of patients accessing services over 1 year (95% CI)** 2			
Community residential (long-stay)^3^	217	0	616
Community psychiatric (acute care)^3^	866	0	2464
Day care services^4^	433	0	616
Hospital out-patient service^4^	8660	0	12320
Primary healthcare – treatment^4^	12990	6160	24640
Psychosocial treatment^4^	8660	12320	12320

Adapted from Bruckner 2011 [14].

1 Service coverage: percentage of patients in the population who are expected to use the service or resource over the course of 1 year.

2 Based on severe cases estimated for a state of high political terror.

3 Inpatient and residential service.

4 Outpatient and day care service.

### Staffing Requirements

For the six populations we estimate a need of 154 FTE, or 12.5 FTE per 100,000 of population for meeting the service coverage targets for severe depression and PTSD. This comprises 28 medical staff, 59 nurses and 68 psychosocial care providers (see http://www.qcmhr.uq.edu.au/BODP/ for full breakdown by population). As is common for LMIC’s, the majority of the proposed care is likely to be provided from primary care staff with some mental health training rather than specialist mental health professionals.

### Comparison of Current and Needed Mental Health Service Capacity

Resource and workforce information for Libya was only available for a period 4 years prior to the conflict. [16] Even if these resources remain within the post-conflict environment, our estimates show a shortfall of health workers across all disciplines, which would be required to appropriately respond to the number of cases. WHO estimate there are 0.18 psychiatrists/psychiatric medical officers per 100,000 and this would equate to two medically qualified staff for the six populations. We estimate 28 medical staff are needed for the treatment of severe depression and PTSD in these populations. Similarly, WHO estimates of 0.5 psychiatric nurses per 100,000 of population would equate to six being available for all populations; however, we estimate a total need of 59 psychiatric and general nurses. Perhaps the only group of healthcare workers that appear to meet predicted needs are psychosocial care providers, where psychologists and social workers reportedly total 6.5/100,000 or 80 across the six populations (Table 6).

**Table 6:** Target full-time equivalent staff requirements for the six populations for post-conflict management of severe depression and PTSD cases (including comorbid cases)^1^.

	Population	Medical	Nurses	Psychosocial care providers	Total FTE
Misrata	444,812	10	21	24	54
Benghazi	674,094	15	31	36	82
Tripoli	49,000	1	3	3	7
Misrata displaced	25,000	1	1	2	4
Zintan	40,000	1	2	3	6
Ras Jdir	3,700	0	0	0	0
Total	1,236,606	28	59	68	154

Adapted from Chisholm 2007 [13].

1 Based on severe cases estimated for a state of high political terror.

## Discussion

This is the first study to model the prevalence of selected mental disorders following country-level conflict. In doing so, we draw systematically upon the existing epidemiological research undertaken amongst displaced and conflict-affected populations, published over the past three decades. We have presented the estimated prevalence of depression and PTSD according to levels of population-level political terror, trauma exposure and recency of conflict as they are estimated to have affected six population groups during 2011–2012 Libyan conflict. The statistical modelling reported herein suggest a substantial mental health burden associated with political terror and exposure to traumatic events, reflected in the number of cases of PTSD and depression for exposed populations.

In line with global epidemiological data, depression prevalence is consistently higher than PTSD prevalence across all models. In terms of time effects, our findings demonstrate an overall trend for PTSD prevalence to drop markedly with time since the end of a conflict whilst a reduction in depression prevalence is much more reliant on a reduction in trauma exposure.

Population prevalence estimates of PTSD and depression derived for six population groups identified from areas affected by periods of intense conflict or mass displacement were in the 30% to 40% range within the post-conflict period. These estimates accord with findings from parallel fields of research. A recent systematic review examining major depressive disorder following terrorist attacks suggested that the risk ranges between 20 and 30% in directly affected victims. [48] Another review examining PTSD prevalence demonstrated a prevalence of 20–29% following human-made disasters. [49] Based on these findings, the estimated prevalence of total cases of PTSD and depression are broadly consistent with the other complex emergency settings.

For the purposes of service planning, we further refined these prevalence estimates by focusing only on those cases that were most likely to be associated with severe levels of impairment by integrating the severity proportions of affective and anxiety disorders recorded by the World Mental Health Surveys. Using this information, severe cases of PTSD were estimated at 9.7%–12.4% and severe depression at 18.3%–19.8% during the immediate post-conflict period. Interpolating these estimates to the 1.24 million Libyans within six population groups identified as being affected by exposure to war trauma and conflict indicates that 123,400 people are likely to have severe PTSD and 228,100 severe depression. Not surprisingly, based upon current WHO estimates, [16] the capacity to address mental health needs of the Libyan population falls exceedingly short of what is likely to be required.

The results of the systematic review and meta-regression that form the basis of the current projections indicate that it is not mass conflict in general but rather the population level of exposure to torture, potentially traumatic events and political terror that are the substantive determinants of PTSD and depression prevalence. For this reason, we have limited the attempt to calculate conflict-related mental health projections to those population groups and geographic regions for which there is some information about the extent of trauma exposure.

For the same reasons, we have not attempted to model the conflict related rates for PTSD and depression for the whole of Libya, as the current body of psychiatric epidemiological research has primarily involved small to medium sized population studies of directly affected populations. It is not clear to what extent these estimates can be scaled up to provide prevalence estimates at a national level.

### Implications for Mental Health Services

There is increasing recognition of the importance of responding in a timely manner to population-level mental health problems following conflict. [50], [51] The mhGAP program and other WHO initiatives [10], [15] identify a combination of medical and social interventions delivered principally within the primary care sector as the model most capable of being scaled up to meet population level need. It is important to note that the proposed scale-up of services is for over a 10 year period and not an immediate response goal. [13].

The ability for resource poor and disrupted health systems to meet population needs is invariably difficult and we have elected to only model severe cases of depression and PTSD. It is recognized this may represent an underestimation of overall need, particularly with regard to psychosocial services for less severe cases. This, combined with the fact that the service requirement model used was developed in countries with much lower prevalence rates than exhibited in our estimates for post-conflict Libya, means our predictions may be conservative; and the actual number of persons requiring service utilisation may be higher.

It is typical for a health system to deteriorate during times of war, with infrastructure becoming degraded and health staff fleeing areas most in need of health services. This would result in even fewer available mental health resources than the WHO pre-conflict estimate for Libya. We have not considered the entire spectrum of mental disorders in our estimations, but rather focused on disorders known to be largely affected by conflict. A comprehensive mental health service would have additional requirements to accommodate the full range of mental disorders.

The importance of Libya returning to a state of peace and stability and a positive post-conflict recovery trajectory for the mental health of the population cannot be overstated. Reducing political terror and trauma is crucial in stabilising prevalence of PTSD and depression. [52] Related to this is the documented evidence that many trauma and adversity induced mental disorders, abate once the immediate threat has resolved with a substantial number of people drawing on their natural resilience. [53] In that regard, it is apparent from our models that time since the end of the conflict has a significant role in the normalising of PTSD estimates but plays a lesser role for depression estimates. This has important implications for program planning.

It was not the aim of this paper to explore specific forms of treatment interventions or program design, however, the mhGAP action programme [15] provides clear recommendations for scaling up care for mental, neurological and substance use disorders in low- and middle-income countries and would be appropriate for mental health programming in the Libyan context. We feel it is important to note that investment in new mental hospitals is not recommended and investments in infrastructure (e.g. acute beds in general hospital) should be paired with commensurate investments in human resources both at the secondary and primary health care level. [54] The post-conflict period will see a heavy reliance on international aid and require a well-coordinated response through tools such as 4W mapping. [55] The first 4W mapping exercise undertaken in December suggests an over-representation of specialised services provided in the initial response and indicates an increase in non-specialised services and community/family support could be more inline with ISAC guidelines. [55].

### Limitations and Future Research Recommendations

The modelling in this study drew on an existing, heterogeneous body of epidemiological research from conflict-affected countries around the world. One limitation is the challenge of validation of instruments for use in post-conflict environments. The variability in prevalence rates found in psychiatric epidemiology following complex emergencies is partially attributable to differences in context, methodology, and exposure to risk factors which have been identified in previously published work. [56] Whilst attempts have been made to account for this variability in our analysis there still remains a significant level of unexplained variability. We have represented this by indicating the wide range of uncertainty around the reported prevalence estimates. Nevertheless, the existing body of post-conflict mental health research provides consistent evidence, as indicated by the meta-analytic findings, of a PTE exposure dose-response association with PTSD and depression that offers useful guidance in projecting needs and possible response models.

It is also important to highlight that measurement errors within the PTS have been demonstrated revealing estimates to be conservatively biased by an absolute order of roughly two. [21] This is a reflection of both the scarcity of information and inherent difficulties in measuring human suffering in quantitative terms.

The modelling process was not able to include torture as a variable despite it’s known significance as a risk factor for mental disorders in post-conflict settings [57], [58]. While widespread torture has been documented in Libya in two recent Amnesty International reports [22], [59] these reports did not provide sufficient detail to estimate the prevalence of this form of abuse within the identified populations. Instead the current analysis applied estimates of country level PTS on the basis of the documented reports of widespread conflict and human rights. We further identified high and intermediate conflict-affected regions whose populations were modelled as being exposed to an intermediate and high level of PTE exposure. These should be seen as estimates only as it is not possible to ascertain the precise level of exposure to PTE’s without detailed epidemiological work amongst the identified populations. Modifications to the populations selected and key parameters chosen (trauma exposure and political terror levels in particular) might be useful if the picture changes as more detailed information becomes available.

It has been necessary to apply surrogate measures in the absence of data for a number of other key areas; ‘affective disorders’ has been used as a proxy for depression in establishing comorbidity rates and ‘anxiety disorder’ severity proportions have been used for PTSD. A lack of statistical power and limitations in carrying out subgroup analysis in the meta-regression modelling necessarily means differences between countries or regions is forfeited and the influence of other environmental [56] and specific cultural factors on prevalence estimates is lost. For example, community-specific recovery processes such as social and family support and/or religion could mitigate the effects of trauma.

Population data for affected Libyan regions is also difficult to ascertain in some cases. Without a public domain for verifying official Libyan census data we relied on various web sources to derive best estimates of numbers of people living in areas exposed to the highest levels of violence and trauma. There is no a priori reason to expect that we have either under- or overestimated population sizes, but it is a source of uncertainty.

The need for epidemiological estimates of mental disorders in conflict and post-conflict regions is essential for more effective program prioritisation and planning. Research is needed to fill knowledge gaps and enhance what we have presented in this paper. High quality epidemiological studies from developing and conflict/post-conflict countries are required in order to obtain more accurate baseline prevalence estimates, more statistical power in modelling prevalence estimates, and a better understanding of how cultural and environmental aspects affect modelling. The potential for applying these models, adapted as necessary, to forecasting prevalence estimates in other conflict-affected populations is ready for exploration. It is hoped that with further research and refining of methodologies the modelling will provide even more useful and accurate projections.

## Conclusion

The findings presented in this paper highlight the potential magnitude of the post-conflict mental health need in Libya, a model that can also be applied to other countries experiencing such conflict. Mental health problems are already surfacing, according to reports from mental health teams on the ground. [29] This is at a time when the Libyan health system is extended, particularly in areas most affected by ongoing violence. [4], [60] The findings also underscore the critical importance of restoring safety and security as imperative for stabilising PTSD and depression prevalence [52] and provide practical information to guide programmers and health service providers.

## Supporting Information

Table S1 **Summary of Studies Included in Post-conflict Meta-regression Analysis.**
(DOC)

Table S2 **Modelled prevalence estimates for PTSD and depression for significant substantive factors and stratified by exposure to political terror scale, potentially traumatic events and time since conflict.**
(DOC)

Checklist S1 **PRISMA Checklist.** For Steel Z, Chey T, Silove D, Marnane C, Bryant RA, et al. (2009) Association of Torture and Other Potentially Traumatic Events With Mental Health Outcomes Among Populations Exposed to Mass Conflict and Displacement. JAMA 302∶537.
(DOC)
