# Supplementary material for: Predicting the Impact of the 2011 Conflict in Libya on Population Mental Health: PTSD and Depression Prevalence and Mental Health Service Requirements
Source: PLoS One. 2012 Jul 13;7(7):e40593. doi: 10.1371/journal.pone.0040593 (PMC3396632; doi:10.1371/journal.pone.0040593)
Supplement: Table S1 — Summary of Studies Included in Post-conflict Meta-regression Analysis. (DOC) [file pone.0040593.s001.doc]

**Table S1: Summary of Studies** Included in Post-conflict Meta-regression Analysis

| **Author** | **Sample Characteristics** | **Sample Size** | **Country Surveyed** | **Reference** |
| --- | --- | --- | --- | --- |
| Ahmad et al., 2008 | Random sample of Kurdistan children and their parents drawn from the general population in Duhok city in Iraqi and Uppsala, Sweden. | 541 | Iraq/Sweden | [1] |
| Alhasnawi et al., 2009 | Nationally representative stratified multistage clustered area probability sample of household residents across Iraq. | 4332 | Iraq | [2] |
| Allden et al, 1996 | Snowball sample of Burmese political dissidents in Thailand. | 104 | Thailand | [3] |
| Al-Naser et al, 2000 | Convenience sample of 195 students from Kuwait University, 108 fire-fighters and 101 full-time government employees from Kuwait city. | 404 | Kuwait | [4] |
| Amowitz et al, 2003 | Random cluster survey of female heads of households in Afghanistan and of households of Afghan refugees living in Pakistan for less than 2 years. | 618 | Afghanistan | [5] |
| Basoglu et al, 2005 | Targeted quota sampling of residents of Belgrade (Serbia and Montenegro), Sarajevo (Bosnia-Herzegovina), Rijeka (Croatia) and Banja Luka (Republic of Srpska, Bosnia-Herzegovina) who had experienced one of the index stressors (combat, torture, internal displacement, refugee experience or aerial bombardment) using mixed snowball sampling strategies. | 1358 | Serbia | [6] |
| Bell et al, 2000 | Random selection of displaced female citizens of Srebrenica from social welfare service responsible for displaced people. | 50 | Bosnia-Herzegovina | [7] |
| Bilanakis et al, 1997 | Random selection of refugees from a camp in Petrovac, Serbia, who had been forced from their homes in Banja Luka, Bosnia-Herzegovina 3-4 months prior to the interview. | 58 | Serbia | [8] |
| Bolton et al, 2002 | Community-based random survey of Rwandan adults in Kanzenze Commune, a town heavily populated with Tutsi prior to 1994. | 368 | Rwanda | [9] |
| Bradley & Tawfiq, 2006 | Retrospective analysis of the records of 97 Kurdish people seeking asylum from Turkey in the UK. They had been referred for medical evaluation of torture allegations by a North London legal practice. | 97 | England | [10] |
| Cardozo et al, 2000 | Multistage random cluster survey of ethnic Albanian households in Kosovo, based upon the 1991 Kosovo census. | 1358 | Kosovo | [11] |
| Cardozo et al, 2003 | Multistage random cluster survey of ethnic Albanian households in Kosovo, based upon the 1991 Kosovo census. | 1399 | Kosovo | [12] |
| Cardozo et al, 2004 | Stratified probabilistic survey of Karenni refugees in 3 Thai-Burmese refugee camps. All household participants interviewed. | 495 | Thailand | [13] |
| Cardozo et al, 2004; Cardozo et al, 2005 | National multistage probability cluster survey of non-disabled Afghan residents. | 699 | Afghanistan | [14, 15] |
| Chung & Seo, 2007 | Snowball sample of North Korean defectors resident in South Korea. | 133 | South Korea | [16] |
| Crescenzi et al, 2002 | Quota sampling of previously imprisoned Tibetan refugees who sought asylum in India between 1991 and 1995 selected from handicraft centers, monasteries, a Refugee Reception Center and a Security Department in Dharamsala. | 76 | India | [17] |
| Dahl et al, 1998 | Consecutive Bosnian women attending a non-governmental humanitarian organization in Bosnia | 209 | Bosnia-Herzegovina | [18] |
| de Jong et al, 2000 | Two stage cluster sample of residents and internally displaced people in Freetown, Sierra Leone. | 245 | Sierra Leone | [19] |
| de Jong et al, 2000 | Stratified random sample of refugees in 4 refugee camps near the Tanzanian-Rwandan border. | 854 | Tanzania | [20] |
| de Jong et al, 2001 | Random sampling of inhabitants of a welfare center for internally displaced Sri Lankans using the list of names provided by the camp organizers. | 163 | Sri Lanka | [21, 22] |
| de Jong et al, 2001; de Jong et al, 2003; Punamaki et al, 2005 | Random survey of households in Algeria; three provinces in Cambodia (Batambang, Kampong Speu and Phnom Penh); displaced persons from two temporary shelters in Addis Ababa, Ethiopia; refugee camps, cities and resettlement areas in Gaza. | 3048 | Algeria/Cambodia/Ethiopia/Palestine | [23-25] |
| de Jong et al, 2006 | A multi-stage random cluster design, involving 30 randomly selected Kashmiri villages in India, within which 17 houses were randomly selected and one adult member of each household was randomly selected for participation. | 510 | Kashmir | [26] |
| De Jong et al, 2007 | Surveys were carried out in Chechnya and Ingushetia using a systematic sampling system to obtain a representative group. | 539 | Chechnya | [27] |
| Doney, 1998 | Survey of tortured ex-political prisoners in India recruited through a support group. | 160 | India | [28] |
| Drozdek, 1997 | Survey of 120 male Bosnian refugees arriving in the Netherlands who had been in Serbian concentration camps. | 120 | Netherlands | [29] |
| Ebata & Miyake, 1989 | A survey of Vietnamese refugees resident in transit and processing camps located in Japan. | 152 | Japan | [30] |
| Eytan et al, 2004 | Random selection of ethnic Albanian households from a comprehensive register of repatriated refugees in Kosovo. | 996 | Kosovo | [31] |
| Eytan et al., 2007 | Consecutive asylum seekers presenting for mandatory health screening in Geneva. | 101 | Switzerland | [32] |
| Farhood et al, 1993 | Small area sampling procedure to randomly select 540 families in West Beirut, Lebanon. | 1159 | Lebanon | [33] |
| Farhood et al, 2006 | Random sample of permanent residents in one of two southern Lebanese towns. Only residents who resided for at least 2 years during Israeli occupation, 1978 and 2000. | 256 | Lebanon | [34] |
| Field & Chhim, 2008 | Nonrandom sampling in which participants were selected from strata based on demographic variables in areas serviced by TPO. | 130 | Cambodia | [35] |
| Fox & Tang, 2000 | Every 3rd person on a list of adult Sierra Leonean refugees at the Red Cross in The Gambia, West Africa. | 55 | Gambia | [36] |
| Franciskovic et al., 2008 | Random sample of adults selected by location exposed to one or more war-related traumatic events from 3 Croatian counties. | 727 | Croatia | [37] |
| Gerritsen et al, 2006; Gerritsen et al, 2004 | Random survey of Somali, Afghan and Iranian refugee groups and asylum seekers in the Netherlands. | 410 | Netherlands | [38, 39] |
| Gillespie et al, 2000 | Convenience sample of Malawian returnees residing in, or attending, a social welfare center. | 74 | Malawi | [40] |
| Good et al, 2006 | Indonesian men and women from the regions of Pidie, Bireuen and Aceh Utara were randomly asked to participate. | 596 | Indonesia | [41] |
| Hagengimana et al, 2003  Hashemian et al, 2006 | Random selection of members of a Rwandan Widows' Association who had lost their husband during the genocide were invited to participate. | 100 | Rwanda | [42] |
|  | Probabilistic survey of Iranian civilian residents who had lived in Rabat, Sardasht and Oshnaviyeh, Northwestern Iran, during the Iran-Iraq war. | 153 | Iran | [43] |
| Herrera Rivera et al., 2008 | A cross-sectional study of civilians with a conflict related disability in Guatemala selected from four civilian organizations. | 99 | Guatemala | [44] |
| Hunt & Gakenyi 2005 | Convenience sample of Bosnians obtained through unspecified Bosnian organizations Bosnian refugees resettled to the UK, obtained via refugee groups in the UK. | 190 | Bosnia-Herzegovina/United Kingdom | [45] |
| Igreja et al., 2006 | Convenience sample of inhabitants of two villages in Mozambique. | 91 | Mozambique | [46] |
| Johnson et al., 2008 | A cross-sectional, population-based, multistage random cluster survey of 1666 adults aged 18 years or older. | 1666 | Liberia | [47] |
| Jukic et al, 1997 | Random sample of former prisoners held in a Serbian detention camp at Sremska Mitrovica, who were psychiatrically observed after their release from a Clinic for Infectious Diseases in Zagreb. | 82 | Croatia | [48] |
| Kalafi et al, 2002 | Random selection of male Afghan refugees and residents of Shiraz, Iran. | 82 | Iran | [49] |
| Kagee & Kagee, 2005; Kagee, 2006 | Convenience sample of South African political activists detained by security forces during the apartheid era. | 148 | South Africa | [50, 51] |
| Karam, 1997 | Random sample of Lebanese citizens from one of 4 communities exposed to the war. | 234 | Lebanon | [52] |
| Karam et al, 1998; Karam et al, 1997 | Multi-stage probability cluster sample of selected residential areas in the Bejjeh, Kornet Shehwan, Ashrafieh and Ain Remmaneh regions of Lebanon. | 658 | Lebanon | [52, 53] |
| Karam et al, 2006 | Nationally representative stratified multistage clustered area probability sample of household residents across Lebanon. | 2857 | Lebanon | [54] |
| Karunakara et al, 2004 | Multi-stage probability household sample used to identify households of Ugandans and Sudanese in the West Nile. | 3323 | Uganda/Sudan | [55] |
| Kashdan et al., 2009 | Multi-stage probability cluster survey the general population exposed to war-related traumatic experiences in 1998 and 1999. | 174 | Kosovo | [56] |
| Kim et al., 2007 | Systematic random sampling of 6 registered internally displaced persons camps in Nyala District, Sudan. | 1274 | Sudan | [57] |
| Klaric et al., 2007 | Small area cluster survey of women and residents of four randomly selected city blocks in Western Mostar and Western Herzegovina. | 367 | Bosnia-Herzegovina | [58] |
| Kozaric-Kovacic et al, 2000 | Systematic survey of every second person on the register of displaced persons in several refugee camps situated near Zagreb, Croatia. | 368 | Croatia | [59] |
| Laban et al, 2004 | Stratified random sample of Iraqi asylum seekers residing in the Netherlands. | 294 | Netherlands | [60] |
| Lee et al, 2001 | Convenience sample of North Korean refugees via community organizations providing assistance to refugees. | 170 | China | [61] |
| Mangoud, 1996 | A two-stage sampling procedure of Bosnian and Croatian refugees who arrived within 6 months of the invasion and resident in refugee camps in Split or Zagreb. | 1156 | Croatia | [62] |
| Masmas et al., 2008 | Survey of a cohort of Asylum seekers resident at Center Sandholm . | 142 | Denmark | [63] |
| Michultka et al, 1998 | Convenience sample of central American refugees from various countries, recruited from two social services agencies. | 50 | USA | [64] |
| Modvig et al, 2000 | Survey of 1033 households across 13 districts of East Timor. | 1033 | East Timor | [65] |
| Mofidi et al., 2008 | Multi-stage probability cluster survey of the population of Sannandaj City. | 996 | Iran | [66] |
| Mollica et al, 1993; Mollica et al, 1998 | Multi-stage area probability sampling of Cambodian refugees in the Site 2 refugee camp on the Thai-Cambodian border. | 993 | Thailand | [67, 68] |
| Mollica et al, 1999 | Total population survey of Bosnian refugees living in the Varazadian camp in north-eastern Croatia. | 534 | Croatia | [69] |
| Moreno & Gibbons, 2002 | Snowball sample of Kosovo Albanian caregivers who were refugees in Macedonia. | 79 | Macedonia | [70] |
| Morina & Ford, 2008 | Random small area cluster survey of an urban and rural site in Kosovo. | 102 | Kosovo | [71] |
| Morina et al., 2008 | Random small area cluster survey of geographical site in Kosovo. | 84 | Kosovo | [72] |
| Obilom & Thacher, 2008 | Multistage cluster-sample of eight wards where rioting had occurred during 2008. | 281 | Nigeria | [73] |
| Onyut et al., 2009 | A total population census of Somalis residents and cluster sample of Rwandan residents in Nakivale refugee settlement in South Western Uganda. | 1422 | Uganda | [74] |
| Oruc et al, 2004 | Random survey of women from a community social service center in Sarajevo who had experienced war trauma. | 100 | Bosnia-Herzegovina | [75] |
| Paker et al, 1992 | Total sample of Turkish prisoners who had been imprisoned for at least 6 months. | 246 | Turkey | [76] |
| Peltzer, 1997 | Tortured Malawian ex-prisoners and ex-political detainees identified by snowball sampling. | 180 | Malawi | [77] |
| Peltzer, 1999 | Sudanese refugee community residents in Northern Ugandan camps. No information of sampling frame provided by authors. | 100 | Uganda | [78] |
| Pham et al, 2004 | Multistage, stratified cluster random sample of households in Ngoma, Mabanza, Buyoga and Mutura provinces, Rwanda. | 2091 | Rwanda | [79] |
| Pillay, 2000 | Participants were South African victims of apartheid who voluntarily took part in a truth and reconciliation commission. | 147 | South Africa | [80] |
| Rasmussen et al., 2007 | A representative sample of plaintiffs against the Indian Government in Punjab. | 116 | India | [81] |
| Redwood-Campbell et al., 2008; Redwood-Campbell et al., 2003 | A random sample of Kosovar families evacuated to Hamilton, Canada. | 157 | Canada | [82, 83] |
| Renner et al., 2009; Renner et al., 2006 | A sample of Chechnyan, Afghnai and West African asylum seekers resident in Australia. No information provided by authors on sampling. | 150 | Austria | [84, 85] |
| Reppesgaard, 1997 | Random sample of Tamil villagers in refugee camps in Jaffna and Colombo City, Sri Lanka. | 551 | Sri Lanka | [86] |
| Roberts et al., 2008; Roberts et al., 2009. | Multi-stage, random cluster survey in the town of Juba, the capital of Southern Sudan and of adult IDPs in Gulu and Amuru districts of northern Uganda. | 2452 | Uganda/Sudan | [87, 88] |
| Rosner et al, 2003 | Multistage probability cluster sample of Bosnian households in Sarajevo. | 98 | Bosnia-Herzegovina | [89] |
| Roth, et al 2006; Roth & Ekblad, 2006 | One in five sample of Kosovar Albanians mass evacuated to Sweden during June 1999. | 218 | Sweden | [90, 91] |
| Saab et al, 2003 | Lebanese ex-hostages of war released between 1990-1996. A random sample of a list obtained from a non-governmental organization supporting ex-detainees. | 118 | Lebanon | [92] |
| Sabin et al, 2003 | Household survey of Guatemalan Mayan from five refugee camps in Mexico, selected to approximate the broader Guatemalan refugee population . | 170 | Mexico | [93] |
| Sabin et al, 2006 | Multi-stage probability survey of Guatemalan Mayan households from five remote highland refugee repatriation villages using probability proportional to size. | 179 | Guatemala | [94] |
| Sachs et al., 2008 | Cohort survey of Tibetans over the age of 15 residing at the Tibetan Refugee Reception Center (TRC) Dharamsala, India. | 769 | India | [95] |
| Savjak, 2000 | Participants were in the Serbian republic selected through an employment program. | 229 | Bosnia-Herzegovina | [96] |
| Savjak, 2007 | Random selection of unemployed residents undertaken by Republika Srpska Employment Bureaus. | 229 | Bosnia-Herzegovina | [97] |
| Scholte et al, 2004 | Multistage probability cluster survey of all adult household members in Nangarhar province in Eastern Afghanistan. | 1011 | Afghanistan | [98] |
| Seino et al., 2008 | Mothers of children less than 5 years old, randomly selected from 1400 households in Kabul Province, Afghanistan. | 1172 | Afghanistan | [99] |
| Silove et al., 2006; Silove et al., 2007 | Sample of asylum seekers recruited from random selection of migration agents in Sydney. | 73 | Australia | [100, 101] |
| Smith, Perrin et al, 2001 | Stratified sampling of children in schools in the Mostar area of Bosnia. The mothers of these children were then asked to participate in the study. | 339 | Bosnia-Herzegovina | [102] |
| Somasundaram & Sivayokan, 1994 | Random sample from a pool of 1322 families living in the Kokuvil-East Primary Health Worker area in northern Sri Lanka. One member of each household selected at random for interview. | 98 | Sri Lanka | [103] |
| Steel et al, 2006 | Mixed snowball sample of Mandaean refugees in Sydney, Australia. | 241 | Australia | [104] |
| Stein et al., 2008; Kaminer et al., 2008 | Nationally representative stratified multistage clustered area probability sample of household residents across South Africa. | 4351 | South Africa | [105, 106] |
| Tang & Fox, 2001 | Random selection from Refugee camp listings in Gambia, Senegal. | 80 | Gambia | [107] |
| Terheggen et al, 2001 | Random selection of inhabitants at an adult school in a refugee camp in northern India for Tibetan refugees. All arriving refugees were enrolled in the school. | 76 | India | [108] |
| Thabet et al., 2008 | Cluster sample of households from two villages, one camp, and one city area exposed to regular shelling. | 200 | Palestine | [109] |
| Thapa & Hauff 2005 | Household survey of internally displaced persons in Nepal living around the UN district headquarters. | 290 | Nepal | [110] |
| Thulesius & Hakansson, 1999 | Consecutive cohort of newly arrived Bosnian refugee residents in an asylum center in Sweden. | 206 | Sweden | [111] |
| Toscani et al, 2007 | Random sample of repatriated ethnic Kosovar Albanians who had received asylum in Switzerland between August 1999 and July 2001. | 580 | Serbia | [112] |
| Van Ommeren et al, 2001 | Random sample of a population of 2331 survivors of physical torture registered by the Center for the Victims of Torture in the UN refugee camp and a matched control sample of non-tortured refugees in Terai in eastern Nepal. | 810 | Nepal | [113] |
| Vinck, 2007 | A multi-stage probability sample of the Gulu, Kitgum, Lira and Soroti regions in northern Uganda with proportional to size sampling of refugee camps for internally displaced persons. | 2585 | Uganda | [114] |
| Yeomans et al., 2008 | Convenience sample of rural Burundian women with a trauma history. | 78 | Burundi | [115] |
| Zlotnick et al., 2006 | Nationally representative stratified multistage clustered area probability sample of household residents across Chile. | 2390 | Chile | [116] |
| Zungu-Dirwayi et al, 2004 | Volunteer and snowball sample of South African in the Western Cape region who had experienced a gross violation of human rights. | 134 | South Africa | [117] |

**References:**

1. Ahmad A, Von Knorring A-L, Sundelin-Wahlsten V. Traumatic experiences and post-traumatic stress disorder in Kurdistanian children and their parents in homeland and exile: an epidemiological approach. *Nordic Journal of Psychiatry.* 2008;62(6):457-463.
2. Alhasnawi S, Sadik S, Rasheed M, Baban A, Al-Alak MM, Othman AY, Ismet N, Shawani O, Murthy S, AlJadiry M, Chatterji S, Al-Gasseer N, Streel E, Naidoo N, Ali MM, Gruber MJ, Petukhova M, Sampson NA, Kessler RC. The prevalence and correlates of DSM-IV disorders in the Iraq Mental Health Survey (IMHS). *World Psychiatry.* 2009;8(2):1-13.
3. Allden K, Poole C, Chantavanich S, Ohmar K, Aung NN, Mollica RF. Burmese political dissidents in Thailand: trauma and survival among young adults in exile. *American Journal of Public Health.* 1996;86(11):1561-1569.
4. al-Naser F, al-Khulaifi IM, Martino C. Assessment of posttraumatic stress disorder four and one-half years after the Iraqi invasion. *International Journal of Emergency Mental Health.* 2000;2(3):153-156.
5. Amowitz LL, Heisler M, Iacopino V. A population-based assessment of women's mental health and attitudes toward women's human rights in Afghanistan. *Journal of Women's Health.* 2003;12(6):577-587.
6. Basoglu M, Livanou M, Crnobaric C, Franciskovic T, Suljic E, Duric D, Vranesic M. Psychiatric and cognitive effects of war in former Yugoslavia: association of lack of redress for trauma and posttraumatic stress reactions. *JAMA.* 2005;294(5):580-590.
7. Bell P, Bergeret I, Oruc L. Women from the safe haven: The psychological and psychiatric consequences of extreme and prolonged trauma on women from Srebrenica. Paper presented at: The psychosocial consequences of war: Results of empirical research from the territory of the former Yugoslavia, 2000; Sarajevo.
8. Bilanakis N, Pappas E, Baldic V, Jokic M. Post-traumatic stress disorder in a refugee camp in Serbia. *Torture.* 1997;7(1):17-20.
9. Bolton P, Neugebauer R, Ndogoni L. Prevalence of depression in rural Rwanda based on symptom and functional criteria. *Journal of Nervous and Mental Disease.* Sep 2002;190(9):631-637.
10. Bradley L, Tawfiq N. The physical and psychological effects of torture in Kurds seeking asylum in the United Kingdom. *Torture.* 2006;16(1):41-47.
11. Cardozo BL, Vergara A, Agani F, Gotway CA. Mental health, social functioning, and attitudes of Kosovar Albanians following the war in Kosovo. *JAMA.* 2000;284(5):569-577.
12. Cardozo BL, Kaiser R, Gotway CA, Agani F. Mental health, social functioning, and feelings of hatred and revenge of Kosovar Albanians one year after the war in Kosovo. *Journal of Traumatic Stress.* 2003;16(4):351-360.
13. Cardozo BL, Talley L, Burton A, Crawford C. Karenni refugees living in Thai-Burmese border camps: traumatic experiences, mental health outcomes, and social functioning. *Social Science & Medicine.* 2004;58(12):2637-2644.
14. Cardozo BL, Bilukha OO, Crawford CAG, Shaikh I, Wolfe MI, Gerber ML, Anderson M. Mental health, social functioning, and disability in postwar Afghanistan. *JAMA.* 2004;292(5):575-584.
15. Cardozo BL, Bilukha OO, Gotway CA, Wolfe MI, Gerber ML, Anderson M. Report from the CDC: mental health of women in postwar Afghanistan. *Journal of Women's Health.* 2005;14(4):285-293.
16. Chung S, Seo J-Y. A study on posttraumatic stress disorder among North Korean defectors and their social adjustment in South Korea. *Journal of Loss and Trauma.* 2007;12(4):365-382.
17. Crescenzi A, Ketzer E, Van Ommeren M, Phuntsok K, Komproe I, de Jong JTVM. Effect of political imprisonment and trauma history on recent Tibetan refugees in India. *Journal of Traumatic Stress.* 2002;15(5):369-375.
18. Dahl S, Mutapcic A, Schei B. Traumatic events and predictive factors for posttraumatic symptoms in displaced Bosnian women in a war zone. *Journal of Traumatic Stress.* 1998;11(1):137-145.
19. de Jong K, Mulhern M, Ford N, van der Kam S, Kleber R. The trauma of war in Sierra Leone. *Lancet.* 2000;355(9220):2067-2068.
20. de Jong JP, Scholte WF, Koeter MW, Hart AA. The prevalence of mental health problems in Rwandan and Burundese refugee camps. *Acta Psychiatrica Scandinavica.* 2000;102(3):171-177.
21. de Jong K, Mulhern M, Ford N, Simpson I, Swan A, Van der Kam S. Psychological trauma of the civil war in Sri Lanka. *Lancet.* 2002;359(9316):1517-1518.
22. de Jong K, Mulhern M, Swan A, van der Kam S. *Assessing trauma in Vavuniya, Sri Lanka.* Amsterdam, the Netherlands: Medicins Sans Frontieres; 2001.
23. de Jong JT, Komproe IH, Van Ommeren M. Common mental disorders in postconflict settings. *Lancet.* 2003;361(9375):2128-2130.
24. de Jong JT, Komproe IH, Van Ommeren M, El Masri M, Araya M, Khaled N, van De Put W, Somasundaram D. Lifetime events and posttraumatic stress disorder in 4 postconflict settings. *JAMA.* 2001;286(5):555-562.
25. Punamaki R-L, Komproe IH, Qouta S, Elmasri M, de Jong JTVM. The role of peritraumatic dissociation and gender in the association between trauma and mental health in a Palestinian community sample. *American Journal of Psychiatry.* 2005;162(3):545-551.
26. De Jong K, Van der Kam S, Fromm S, van Galen R, Kemmere T, van der Weerd H, Ford N, Hayes L. *Kashmir: Violence and health.* Amsterdam, the Netherlands: Medicins Sans Frontieres; 2006.
27. de Jong K, van der Kam S, Ford N, Hargreaves S, van Oosten R, Cunningham D, Boots G, Andrault E, Kleber RJ. The trauma of ongoing conflict and displacement in Chechnya: Quantitative assessment of living conditions, and psychosocial and general health status among war displaced in Chechnya and Ingushetia. *Conflict and Health.* 2007;1(4).
28. Doney A. The psychological after-effects of torture: a survey of Sri Lankan ex-detainees. In: Somasundaram D, ed. *Scarred minds: the psychological impact of war on Sri Lankan Tamils*. New Delhi: Sage Publications; 1997:256-287.
29. Drozdek B. Follow-up study of concentration camp survivors from Bosnia-Herzegovina: three years later. *Journal of Nervous & Mental Disease.* Nov 1997;185(11):690-694.
30. Ebata K, Miyake Y. A mental health survey of the Vietnamese refugee in Japan. *International Journal of Social Psychiatry.* 1989;35(2):164-172.
31. Eytan A, Gex-Fabry M, Toscani L, Deroo L, Loutan L, Bovier PA. Determinants of postconflict symptoms in Albanian Kosovars. *Journal of Nervous & Mental Disease.* 2004;192(10):664-671.
32. Eytan A, Durieux-Paillard S, Whitaker-Clinch B, Loutan L, Bovier PA. Transcultural validity of a structured diagnostic interview to screen for major depression and posttraumatic stress disorder among refugees. *Journal of Nervous & Mental Disease.* 2007;195(9):723-728.
33. Farhood L, Zurayk H, Chaya M, Saadeh F, Meshefedjian G, Sidani T. The impact of war on the physical and mental health of the family: the Lebanese experience. *Social Science & Medicine.* 1993;36(12):1555-1567.
34. Farhood L, Dimassi H, Lehtinen T. Exposure to war-related traumatic events, prevalence of PTSD, and general psychiatric morbidity in a civilian population from Southern Lebanon. *Journal of Transcultural Nursing.* 2006;17(4):333-340.
35. Field NP, Chhim S. Desire for revenge and attitudes toward the Khmer Rouge Tribunal Among Cambodians. *Journal of Loss & Trauma.* 2008;13(4):352-372.
36. Fox SH, Tang SS. The Sierra Leonean refugee experience: traumatic events and psychiatric sequelae. *Journal of Nervous & Mental Disease.* 2000;188(8):490-495.
37. Franciskovic T, Tovilovic Z, Sukovic Z, Stevanovic A, Ajdukovic D, Kraljevic R, Bogic M, Priebe S. Health care and community-based interventions for war-traumatized people in Croatia: community-based study of service use and mental health. *Croatian Medical Journal.* 2008;49(4):483-490.
38. Gerritsen AA, Bramsen I, Deville W, van Willigen LH, Hovens JE, van der Ploeg HM. Physical and mental health of Afghan, Iranian and Somali asylum seekers and refugees living in the Netherlands. *Social Psychiatry and Psychiatric Epidemiology.* 2006;41(1):18-26.
39. Gerritsen AAM, Bramsen I, Deville W, van Willigen LHM, Hovens JE, van der Ploeg HM. Health and health care utilisation among asylum seekers and refugees in the Netherlands: design of a study. *BMC Public Health.* 2004;4:7.
40. Gillespie A, Peltzer K, MacLachlan M. Returning refugees: Psychosocial problems and mediators of mental health among Malawian returnees. *Journal of Mental Health.* 2000;9(2):165-178.
41. Good B, DelVecchio Good M-J, Grayman J, Lakoma M. *A psychosocial needs assessment of communities affected by the conflict in the districts of Pidie, Bireuen and Aceh Utara.* Banda Aceh: International Organization for Migration (IOM) and the Department of Social Medicine from Harvard Medical School; 2006.
42. Hagengimana A, Hinton D, Bird B, Pollack M, Pitman RK. Somatic panic-attack equivalents in a community sample of Rwandan widows who survived the 1994 genocide. *Psychiatry Research.* 2003;117(1):1-9.
43. Hashemian F, Khoshnood K, Desai MM, Falahati F, Kasl S, Southwick S. Anxiety, depression, and posttraumatic stress in Iranian survivors of chemical warfare. *JAMA.* 2006;296(5):560-566.
44. Herrera Rivera W, Mari JdJ, Andreoli SB, Quintana MI, Ferraz MPdT. Prevalence of mental disorder and associated factors in civilian Guatemalans with disabilities caused by the internal armed conflict. *International Journal of Social Psychiatry.* 2008;54(5):414-424.
45. Hunt N, Gakenyi M. Comparing refugees and nonrefugees: the Bosnian experience. *Journal of Anxiety Disorders.* 2005;19(6):717-723.
46. Igreja V, Kleijn W, Richters A. When the war was over, little changed: women's posttraumatic suffering after the war in Mozambique. *Journal of Nervous & Mental Disease.* 2006;194(7):502-509.
47. Johnson K, Asher J, Rosborough S, Raja A, Panjabi R, Beadling C, Lawry L. Association of combatant status and sexual violence with health and mental health outcomes in postconflict Liberia. *JAMA.* 2008;300(6):676-690.
48. Jukic V, Dodig G, Kenfelj H, De Zan D. Psychical difficulties in former prisoners of detention camps. *Collegium Antropologicum.* 1997;21(1):235-242.
49. Kalafi Y, Hagh-Shenas H, Ostovar A. Mental health among Afghan refugees settled in Shiraz, Iran. *Psychological Reports.* 2002;90(1):262-266.
50. Kagee A. Symptoms of distress and posttraumatic stress among South African former political detainees. *Ethnicity & Health.* 2005;10(2):169-179.
51. Kagee A. The relationship between statement giving at the South African Truth and Reconciliation Commission and psychological distress among former political detainees. *South African Journal of Psychology.* 2006;36(1):10-24.
52. Karam EG. Comorbidity of posttraumatic stress disorder and depression. In: Fullerton CS, Ursano R, eds. *Posttraumatic stress disorder: Acute and long-term responses to trauma and disaster*. Washington, DC: American Psychiatric Association.; 1997:xii, 296.
53. Karam EG, Howard DB, Karam AN, Ashkar A, Shaaya M, Melhem N, El-Khoury N. Major depression and external stressors: the Lebanon Wars. *European Archives of Psychiatry & Clinical Neuroscience.* 1998;248(5):225-230.
54. Karam EG, Mneimneh ZN, Karam AN, Fayyad JA, Nasser SC, Chatterji S, Kessler RC. Prevalence and treatment of mental disorders in Lebanon: a national epidemiological survey. *Lancet.* 2006;367(9515):1000-1006.
55. Karunakara UK, Neuner F, Schauer M, Singh S, Hill K, Elbert T, Burnha G. Traumatic events and symptoms of post-traumatic stress disorder amongst Sudanese nationals, refugees and Ugandans in the West Nile. *African Health Sciences.* 2004;4(2):83-93.
56. Kashdan TB, Morina N, Priebe S. Post-traumatic stress disorder, social anxiety disorder, and depression in survivors of the Kosovo War: experiential avoidance as a contributor to distress and quality of life. *Journal of Anxiety Disorders.* 2009;23(2):185-196.
57. Kim G, Torbay R, Lawry L. Basic health, women's health, and mental health among internally displaced persons in Nyala Province, South Darfur, Sudan. *American Journal of Public Health.* 2007;97(2):353-361.
58. Klaric M, Klaric B, Stevanovic A, Grkovic J, Jonovska S. Psychological consequences of war trauma and postwar social stressors in women in Bosnia and Herzegovina. *Croatian Medical Journal.* 2007;48(2):167-176.
59. Kozaric-Kovacic D, Ljubin T, Grappe M. Comorbidity of posttraumatic stress disorder and alcohol dependence in displaced persons. *Croatian Medical Journal.* 2000;41(2):173-178.
60. Laban CJ, Gernaat HBPE, Komproe IH, Schreuders BA, De Jong JTVM. Impact of a long asylum procedure on the prevalence of psychiatric disorders in Iraqi asylum seekers in The Netherlands. *Journal of Nervous & Mental Disease.* 2004;192(12):843-851.
61. Lee Y, Lee MK, Chun KH, Lee YK, Yoon SJ. Trauma experience of North Korean refugees in China. *American Journal of Preventive Medicine.* 2001;20(3):225-229.
62. Mangoud AM. War related stress disorder among Bosnian and Croatian refugees. *Saudi Medical Journal.* 1996;17(2):205-211.
63. Masmas TN, Møller E, Buhmannr C, Bunch V, Jensen JH, Hansen TN, Jørgensen LM, Kjaer C, Mannstaedt M, Oxholm A, Skau J, Theilade L, Worm L, Ekstrøm M. Asylum seekers in Denmark: a study of health status and grade of traumatization of newly arrived asylum seekers. *Torture.* 2008;18(2):77-86.
64. Michultka D, Blanchard EB, Kalous T. Responses to civilian war experiences: predictors of psychological functioning and coping. *Journal of Traumatic Stress.* 1998;11(3):571-577.
65. Modvig J, Pagaduan-Lopez J, Rodenburg J, Salud CM, Cabigon RV, Panelo CI. Torture and trauma in post-conflict East Timor. *Lancet.* 2000;356(9243):1763.
66. Mofidi N, Ghazinour M, Araste M, Jacobsson L, Richter J. General mental health, quality of life and suicide-related attitudes among Kurdish people in Iran. *International Journal of Social Psychiatry.* 2008;54(5):457-468.
67. Mollica RF, Donelan K, Tor S, Lavelle J, Elias C, Frankel M, Blendon RJ. The effect of trauma and confinement on functional health and mental health status of Cambodians living in Thailand-Cambodia border camps. *JAMA.* 1993;270(5):581-586.
68. Mollica RF, McInnes K, Poole C, Tor S. Dose-effect relationships of trauma to symptoms of depression and post-traumatic stress disorder among Cambodian survivors of mass violence. *British Journal of Psychiatry.* 1998;173:482-488.
69. Mollica RF, McInnes K, Sarajlic N, Lavelle J, Sarajlic I, Massagli MP. Disability associated with psychiatric comorbidity and health status in Bosnian refugees living in Croatia. *JAMA.* 1999;282(5):433-439.
70. Moreno IT, Gibbons JL. Trauma events, residence in refugee camps, and educational attainment as predictors of trauma symptoms of Albanian refugees in Macedonia. *International Journal of Group Tensions.* 2002;31(2):155-174.
71. Morina N, Ford JD. Complex sequelae of psychological trauma among Kosovar civilian war victims. *International Journal of Social Psychiatry.* Sep 2008;54(5):425-436.
72. Morina N, Stangier U, Risch AK. Experiential avoidance in civilian war survivors with current versus recovered posttraumatic stress disorder: A pilot study. *Behaviour Change.* 2008;25(1):15-22.
73. Obilom RE, Thacher TD. Posttraumatic stress disorder following ethnoreligious conflict in Jos, Nigeria. *Journal of Interpersonal Violence.* 2008;23(8):1108-1119.
74. Onyut LP, Neuner F, Ertl V, Schauer E, Odenwald M, Elbert T. Trauma, poverty and mental health among Somali and Rwandese refugees living in an African refugee settlement - an epidemiological study. *Conflict & Health.* 2009;3:6.
75. Oruc L, Kapur L, Pojski N, Cavaljuga S, Ivezi S, Bell P. Posttraumatic stress disorder among women after the war in Sarajevo: a rationale for genetic study. *Bosnian Journal of Basic Medical Sciences.* 2004;4(1):50-52.
76. Paker M, Paker O, Yuksel S. Psychological effects of torture: An empirical study of tortured and non-tortured non-political prisoners. In: Basoglu M, ed. *Torture and its consequences: Current treatment approaches*. New York, NY: Cambridge University Press.; 1992:xxiii, 527.
77. Peltzer K. Psychological effects of torture: a comparison of political detainees and non-political prisoners in Malawi. *Torture.* 1997;7(2):48-53.
78. Peltzer K. Trauma and mental health problems of Sudanese refugees in Uganda. *Central African Journal of Medicine.* 1999;45(5):110-114.
79. Pham PN, Weinstein HM, Longman T. Trauma and PTSD symptoms in Rwanda: implications for attitudes toward justice and reconciliation. *JAMA.* 2004;292(5):602-612.
80. Pillay BJ. Providing mental health services to survivors: a Kwa Zulu-Natal perspective. *Ethnicity and Health.* 2000;5(3/4):269-272.
81. Rasmussen A, Rosenfeld B, Reeves K, Keller AS. The effects of torture-related injuries on long-term psychological distress in a Punjabi Sikh sample. *Journal of Abnormal Psychology.* 2007;116(4):734-740.
82. Redwood-Campbell L, Thind H, Howard M, Koteles J, Fowler N, Kaczorowski J. Understanding the health of refugee women in host countries: lessons from the Kosovar re-settlement in Canada. *Prehospital & Disaster Medicine.* 2008;23(4):322-327.
83. Redwood-Campbell L, Fowler N, Kaczorowski J, Molinaro E, Robinson S, Howard M, Jafarpour M. How are new refugees doing in Canada? Comparison of the health and settlement of the Kosovars and Czech Roma. *Canadian Journal of Public Health.* 2003;94(5):381-385.
84. Renner W, Salem I. Post-traumatic stress in asylum seekers and refugees from Chechnya, Afghanistan, and West Africa: gender differences in symptomatology and coping. *International Journal of Social Psychiatry.* 2009;55(2):99-108.
85. Renner W, Salem I, Ottomeyer K. Cross-cultural validation of measures of traumatic symptoms in groups of asylum seekers from Chechnya, Afghanistan, and West Africa. *Social Behavior and Personality.* 2006;34(9):1101-1114.
86. Reppesgaard HO. Studies on psychosocial problems among displaced people in Sri Lanka. *European Journal of Psychiatry.* 1997;11(4):223-234.
87. Roberts B, Ocaka KF, Browne J, Oyok T, Sondorp E. Factors associated with post-traumatic stress disorder and depression amongst internally displaced persons in northern Uganda. *BMC Psychiatry.* 2008;8:38.
88. Roberts B, Damundu EY, Lomoro O, Sondorp E. Post-conflict mental health needs: a cross-sectional survey of trauma, depression and associated factors in Juba, Southern Sudan. *BMC Psychiatry.* 2009;9:7.
89. Rosner R, Powell S, Butollo W. Posttraumatic Stress Disorder three years after the siege of Sarajevo. *Journal of Clinical Psychology.* 2003;59(1):41-55.
90. Roth G, Ekblad S. A longitudinal perspective on depression and sense of coherence in a sample of mass-evacuated adults from Kosovo. *Journal of Nervous & Mental Disease.* 2006;194(5):378-381.
91. Roth G, Ekblad S, Agren H. A longitudinal study of PTSD in a sample of adult mass-evacuated Kosovars, some of whom returned to their home country. *European Psychiatry.* 2006;21(3):152-159.
92. Saab BR, Chaaya M, Doumit M, Farhood L. Predictors of psychological distress in Lebanese hostages of war. *Social Science & Medicine.* 2003;57(7):1249-1257.
93. Sabin M, Lopes Cardozo B, Nackerud L, Kaiser R, Varese L. Factors associated with poor mental health among Guatemalan refugees living in Mexico 20 years after civil conflict. *JAMA.* 2003;290(5):635-642.
94. Sabin M, Sabin K, Kim HY, Vergara M, Varese L. The mental health status of Mayan refugees after repatriation to Guatemala. *Pan American Journal of Public Health.* 2006;19(3):163-171.
95. Sachs E, Rosenfeld B, Lhewa D, Rasmussen A, Keller A. Entering exile: trauma, mental health, and coping among Tibetan refugees arriving in Dharamsala, India. *Journal of Traumatic Stress.* 2008;21(2):199-208.
96. Savjak N. Displacement as a factor causing posttraumatic stress disorder. Paper presented at: The psychosocial consequences of war: Results of empirical research from the territory of former Yugoslavia, 2000; Sarajevo.
97. Savjak N. Multiple traumatisation as a risk factor of post-traumatic stress disorder. In: Trappler B, ed. *Modern terrorism and psychological trauma*. New York, NY: Gordian Knot Books/Richard Altschuler & Associates; 2007:101-112.
98. Scholte WF, Olff M, Ventevogel P, de Vries G-J, Jansveld E, Cardozo BL, Crawford CAG. Mental health symptoms following war and repression in eastern Afghanistan. *JAMA.* 2004;292(5):585-593.
99. Seino K, Takano T, Mashal T, Hemat S, Nakamura K. Prevalence of and factors influencing posttraumatic stress disorder among mothers of children under five in Kabul, Afghanistan, after decades of armed conflicts. *Health & Quality of Life Outcomes.* 2008;6:29.
100. Silove D, Steel Z, Susljik I, Frommer N, Loneragan C, Brooks R, Le Touze D, Manicavasagar V, Coello M, Smith M, Harris E. Torture, mental health status and the outcomes of refugee applications among recently arrived asylum seekers in Australia. *International Journal of Migration, Health and Social Care.* 2006;2(1):4-14.
101. Silove D, Steel Z, Susljik I, Frommer N, Loneragan C, Chey T, Brooks R, le Touze D, Ceollo M, Smith M, Harris E, Bryant R. The impact of the refugee decision on the trajectory of PTSD, anxiety, and depressive symptoms among asylum seekers: a longitudinal study. *American Journal of Disaster Medicine.* 2007;2(6):321-329.
102. Smith P, Perrin S, Yule W, Rabe-Hesketh S. War exposure and maternal reactions in the psychological adjustment of children from Bosnia-Hercegovina. *Journal of Child Psychology and Psychiatry.* 2001;42(3):395-404.
103. Somasundaram DJ, Sivayokan S. War trauma in a civilian population. *British Journal of Psychiatry.* 1994;165(4):524-527.
104. Steel Z, Silove D, Brooks R, Momartin S, Alzuhairi B, Susljik I. Impact of immigration detention and temporary protection on the mental health of refugees. *British Journal of Psychiatry.* 2006;188:58-64.
105. Stein DJ, Seedat S, Herman A, Moomal H, Heeringa SG, Kessler RC, Williams DR. Lifetime prevalence pf psychiatric disorders in South Africa. *British Journal of Psychiatry.* 2008;192(2):112-117.
106. Kaminer D, Grimsrud A, Myer L, Stein DJ, Williams DR. Risk for post-traumatic stress disorder associated with different forms of interpersonal violence in South Africa. *Social Science and Medicine.* 2008;67(10):1589-1595.
107. Tang SS, Fox SH. Traumatic experiences and the mental health of Senegalese refugees. *Journal of Nervous & Mental Disease.* 2001;189(8):507-512.
108. Terheggen MA, Stroebe MS, Kleber RJ. Western conceptualizations and Eastern experience: a cross-cultural study of traumatic stress reactions among Tibetan refugees in India. *Journal of Traumatic Stress.* 2001;14(2):391-403.
109. Thabet AA, Abu Tawahina A, El Sarraj E, Vostanis P. Exposure to war trauma and PTSD among parents and children in the Gaza strip. *European Child & Adolescent Psychiatry.* 2008;17(4):191-199.
110. Thapa SB, Hauff E. Psychological distress among displaced persons during an armed conflict in Nepal. *Social Psychiatry & Psychiatric Epidemiology.* 2005;40(8):672-679.
111. Thulesius H, Hakansson A. Screening for posttraumatic stress disorder symptoms among Bosnian refugees. *Journal of Traumatic Stress.* 1999;12(1):167-174.
112. Toscani L, Deroo LA, Eytan A, Gex-Fabry M, Avramovski V, Loutan L, Bovier P. Health status of returnees to Kosovo: do living conditions during asylum make a difference? *Public Health.* 2007;121(1):34-44.
113. Van Ommeren M, de Jong JT, Sharma B, Komproe I, Thapa SB, Cardena E. Psychiatric disorders among tortured Bhutanese refugees in Nepal. *Archives of General Psychiatry.* 2001;58(5):475-482.
114. Vinck P, Pham PN, Stover E, Weinstein HM. Exposure to war crimes and implications for peace building in northern Uganda. *JAMA.* 2007;298(5):543-554.
115. Yeomans PD, Herbert JD, Forman EM. Symptom comparison across multiple solicitation methods among Burundians with traumatic event histories. *Journal of Traumatic Stress.* 2008;21(2):231-234.
116. Zlotnick C, Johnson J, Kohn R, Vicente B, Rioseco P, Saldivia S. Epidemiology of trauma, post-traumatic stress disorder (PTSD) and co-morbid disorders in Chile. *Psychological Medicine.* 2006;36(11):1523-1533.
117. Zungu-Dirwayi NM, Kaminer DM, Mbanga IR, Stein DJM. The Psychiatric Sequelae of Human Rights Violations: A Challenge for Primary Health Care. *Journal of Nervous & Mental Disease.* 2004;192(4):255-259.
