# Supplementary material for: Predicting the Impact of the 2011 Conflict in Libya on Population Mental Health: PTSD and Depression Prevalence and Mental Health Service Requirements
Source: PLoS One. 2012 Jul 13;7(7):e40593. doi: 10.1371/journal.pone.0040593 (PMC3396632; doi:10.1371/journal.pone.0040593)
Supplement: Table S2 — Modelled prevalence estimates for PTSD and depression for significant substantive factors and stratified by exposure to political terror scale, potentially traumatic events and time since conflict. (DOC) [file pone.0040593.s002.doc]

**Table S2: Modelled prevalence estimates for PTSD and depression for significant substantive factors and stratified by exposure to political terror scale, potentially traumatic events and time since conflict**

|  | % Δ Variance | Odds ratio (95%CI) | Total case prevalence estimate (%) (95%CI) | Severe case prevalence estimate (%) (95%CI) |
| --- | --- | --- | --- | --- |
| **PTSD‡** |  |  |  |  |
| PTE adversity ratio | + 5.8% |  |  |  |
| Moderate (< 0.3) † |  | 1.00 | 20.7 (14.6-28.4) | 6.2 (4.4-8.5) |
| High (≥ 0.3) |  | 2.00 (1.16-3.45)* | 34.3 (23.2-47.4) | 10.3 (7.0-14.2) |
| Time since conflict | + 7.7% |  |  |  |
| ≥ 3 years† |  | 1.00 | 26.6 (20.9-33.2) | 14.2 (6.3-10.0) |
| <3 years |  | 1.87 (1.23-2.85)* | 40.5 (30.9-50.8) | 12.2 (9.3-15.2) |
| Political Terror Scale | +9.2% |  |  |  |
| PTS < 4.0† |  | 1.00 | 24.6 (18.9-31.5) | 7.4 (5.7-9.5) |
| PTS ≥ 4.0 |  | 2.11 (1.36-3.27)* | 40.8 (30.7-51.7) | 12.2 (9.2-15.5) |
| PTS x time since conflict | +13.2% |  |  |  |
| Low political terror*Past conflict† |  | 1.00 | 24.1 (17.6-32.0) | 7.3 (5.3-9.7) |
| Low political terror *Recent conflict |  | 1.19 (0.56-2.51) | 27.4 (15.1-44.3) | 8.3 (4.6-13.4) |
| High political terror * Past conflict |  | 1.25 (0.62-2.51) | 28.3 (16.4-44.4) | 8.6 (4.9-3.5) |
| High political terror * Recent conflict |  | 2.61 (1.58-4.30)* | 45.2 (33.4-57.7) | 13.7 (10.1-17.5) |
| PTE adversity ratio x time since conflict | + 16.8% |  |  |  |
| Moderate trauma * Past conflict † |  | 1.00 | 16.8 (10.7-25.6) | 5.1 (3.2-7.8) |
| High trauma * Past conflict |  | 1.87 (0.82-4.26) | 27.5 (14.3-46.3) | 8.3 (4.3-14) |
| Moderate trauma * Recent conflict |  | 1.55 (0.77-3.13) | 23.9 (13.5-38.8) | 7.2 (4.1-11.8) |
| High trauma * Recent conflict |  | 3.63 (1.87-7.03)* | 42.4 (27.5-58.8) | 12.8 (8.3-17.8) |
| PTS x PTE adversity ratio | + 18.3% |  |  |  |
| Low political terror * Moderate trauma † |  | 1.00 | 18.1 (12.1-26.1) | 5.5 (3.7-7.9) |
| Low political terror * High trauma |  | 1.17 (0.59-2.33) | 20.5 (11.5-34.0) | 6.2 (3.5-10.3) |
| High political terror * Moderate trauma |  | 2.16 (1.01-4.64)* | 32.3 (18.2-50.6) | 9.7 (5.5-15.2) |
| High political terror * High trauma |  | 3.18 (1.79-5.68)* | 41.3 (28.3-55.6) | 12.4 (8.5-16.7) |
| **Depression‡** |  |  |  |  |
| PTE adversity ratio | + 14.9% |  |  |  |
| Moderate (< 0.3) † |  | 1.00 | 20.9 (14.9-28.6) | 11.0 (7.9-15.2) |
| High (≥ 0.3) |  | 2.52 (1.64-3.88)* | 40.0 (30.3-50.6) | 21.2 (16.1-26.8) |
| Time since conflict | + 3.5% |  |  |  |
| ≥ 3 years† |  | 1.00 | 29.6 (22.2-38.4) | 15.7 (11.8-20.4) |
| <3 years |  | 1.60 (1.01-2.51)* | 40.2 (29.9-51.4) | 21.3 (15.8-27.2) |
| Political Terror Scale | +5.5% |  |  |  |
| PTS < 4.0† |  | 1.00 | 26.4 (19.8-34.3) | 14.0 (10.5-18.2) |
| PTS ≥ 4.0 |  | 1.78 (1.11-2.85)* | 38.9 (28.4-50.6) | 20.6 (15.1-26.8) |
| PTS x time since conflict | + 6.6% |  |  |  |
| Low political terror*Past conflict† |  | 1.00 | 27.1 (19.3-36.6) | 14.4 (10.2-19.4) |
| Low political terror *Recent conflict |  | 1.25 (0.58-2.71) | 31.7 (17.7-50.2) | 16.8 (9.4-26.6) |
| High political terror * Past conflict |  | 1.23 (0.48-3.12) | 31.3 (15.2-53.7) | 16.6 (8.1-28.5) |
| High political terror * Recent conflict |  | 1.99 (1.16-3.39)* | 42.5 (30.2-55.7) | 22.5 (16.0-29.5) |
| PTE adversity ratio x time since conflict | + 17.1% |  |  |  |
| Moderate trauma * Past conflict † |  | 1.00 | 16.9 (11.2-24.6) | 9.0 (6.0-13.0) |
| Moderate trauma * Recent conflict |  | 2.05 (0.94-4.46) | 29.4 (16.0-47.6) | 15.6 (8.5-25.2) |
| High trauma * Past conflict |  | 2.93 (1.54-5.60)* | 37.3 (23.8-53.2) | 19.8 (12.6-28.2) |
| High trauma * Recent conflict |  | 3.17 (1.89-5.32)* | 39.2 (27.8-51.9) | 20.8 (14.7-27.5) |
| PTS x PTE adversity ratio | + 15.5% |  |  |  |
| Low political terror * Moderate trauma † |  | 1.00 | 16.8 (11.4-24.0) | 8.9 (6.0-12.7) |
| Low political terror * High trauma |  | 2.31 (1.33-4.02)* | 31.8 (21.1-44.8) | 16.9 (11.2-23.7) |
| High political terror * Moderate trauma |  | 2.61 (1.22-5.56)* | 34.5 (19.8-52.9) | 18.3 (10.5-28.0) |
| High political terror * High trauma |  | 2.95 (1.79-4.87)* | 37.3 (26.5-49.6) | 19.8 (14.0-26.3) |

Abbreviations: % Δ variance = percent change in variance from the methodological baseline model; PTSD = Post-traumatic stress disorder; PTS = political terror scale; Ratio = potentially traumatic events (PTE) adversity ratio; ‌‌Time = time since conflict; † reference category; ‡ All substantive predictor models are adjusted for significant methodological predictors (sample size and type of measure); Low political terror = PTS < 4; High political terror = PTS ≥ 4; Recent conflict = < 3 years since end of conflict; Past conflict = ≥ 3 years since end of conflict; Moderate trauma = PTE adversity ratio < 0.3; High trauma = PTE adversity ratio ≥ 0.3; * Statistically significant OR compared to reference group
